# The acceptability and usability of two HIV self‐test kits among men who have sex with men: a randomised crossover trial

**DOI:** 10.5694/mja2.51641

**Published:** 2022-07-12

**Authors:** Dana YL Lee, Jason J Ong, Kirsty Smith, Muhammad S Jamil, Ruthy McIver, Rebecca Wigan, Kate Maddaford, Anna McNulty, John M Kaldor, Christopher K Fairley, Benjamin Bavinton, Marcus Chen, Eric PF Chow, Andrew E Grulich, Martin Holt, Damian P Conway, Mark Stoove, Handan Wand, Rebecca J Guy

**Affiliations:** ^1^ Central Clinical School Monash University Melbourne VIC; ^2^ Melbourne Sexual Health Centre Alfred Health Melbourne VIC; ^3^ Kirby Institute University of New South Wales Sydney NSW; ^4^ Global HIV, Hepatitis and STI Programmes, World Health Organization Geneva Switzerland; ^5^ Sydney Sexual Health Centre Sydney NSW; ^6^ The University of New South Wales Sydney NSW; ^7^ The Burnet Institute Melbourne VIC

**Keywords:** HIV, Diagnostic tests and procedures, Public health, Sexually transmitted diseases

## Abstract

**Objectives:**

To compare the usability and acceptability of oral fluid‐ and blood‐based HIV self‐test kits among men who have sex with men in Australia.

**Design:**

Randomised crossover trial.

**Setting, participants:**

Gay, bisexual, and other men aged 18 years or older who have sex with men, who attended two metropolitan sexual health clinics in Sydney and Melbourne, 7 January – 10 December 2019.

**Main outcome measures:**

Ease of use of HIV self‐test kits; preferred HIV self‐test type; difficulties encountered during HIV self‐testing.

**Results:**

170 men were recruited (median age, 34 years; interquartile range, 29–43 years); 144 identified as gay (85%), 96 were born outside Australia (57%). Participants were more likely to report the oral fluid HIV self‐test was easy to use than the blood‐based self‐test (oral fluid, 99%; blood, 86%; odds ratio [OR], 3.0; 95% confidence interval [CI], 1.4–6.6). The oral fluid test was preferred by 98 participants (58%; 95% CI, 50–65%), the blood‐based test by 69 (41%; 95% CI, 33–48%). Difficulties with the oral fluid test kit identified by observing nurses included problems placing the buffer solution into the stand (40 of 170 participants, 24%) and not swabbing both gums (23 of 169, 14%); difficulties with the blood‐based test kit included problems filling the device test channel (69 of 170, 41%) and squeezing the finger firmly enough to generate a blood drop (42 of 170, 25%). No participant received an invalid result with the oral fluid self‐test; two of 162 participants (1%) received invalid results with the blood self‐test. After adjusting for age, education level, and ethnic background, characteristics associated with higher odds of using HIV self‐testing in the future were overseas birth (adjusted OR, 3.07; 95% CI, 1.42–6.64), and self‐evaluated ease of use and confidence in using the kits.

**Conclusion:**

It is important to provide options for obtaining both oral fluid‐ and blood‐based HIV self‐tests. The usability and acceptability of both kits were high, but the ease of use and perceived accuracy influenced test kit preference.

**The known**: Stigmatisation, the stress of waiting for results, and costs are barriers to HIV testing that can largely be overcome by self‐testing, but restrictions on availability have limited its uptake in Australia.

**The new**: Both HIV self‐test kits were easy to use, but the oral fluid self‐test was reported as being easier to use, and was preferred to the blood‐based kit by 58% of participants. Despite some difficulties during self‐testing, almost all participants received valid test results.

**The implications**: Access to HIV self‐testing should be expanded. TGA approval of an oral fluid‐based test should be considered in order to provide greater choice.

In Australia, diagnoses of human immunodeficiency virus (HIV) infection in gay, bisexual, and other men who have sex with men are declining, primarily among those born in Australia.^1^ HIV testing is a key prevention strategy,^2^ and the gateway to both treatment for those with positive test results and to pre‐exposure prophylaxis (PrEP) for people with negative results.^3^ However, the frequency of HIV testing among men who have sex with men is inadequate, particularly among those who are not receiving PrEP^4^, ^5^ or were born overseas.^6^ Consequently, late HIV diagnoses remain relatively common; during 2008–2017, 23% of Australian‐born and 37% of East Asia‐born men diagnosed with HIV had CD4+ counts below 350 cells/μL.^7^

Barriers to HIV testing include low perceived risk of infection, the stress of waiting for results, and fear of a positive result.^8^ Men born overseas who have sex with men face additional barriers, particularly those without health insurance, including the cost of HIV testing,^9^ poor awareness of HIV testing locations, cultural stigmatisation associated with HIV diagnoses, and fear of judgement by health care providers.^10^ HIV self‐testing can circumvent some of these barriers, allowing men to undergo private testing, to obtain results rapidly, and to eliminate the need to attend a healthcare facility for testing.

A randomised controlled trial (RCT) in Australia found that providing free oral fluid‐based HIV self‐test kits to men who have sex with men doubled the frequency of HIV testing, and almost quadrupled the rate for infrequent testers (those not tested for HIV during the preceding two years).^11^ In November 2018, the Therapeutic Goods Administration (TGA) approved a blood‐based HIV self‐test, the first for an infectious disease approved in Australia.^12^ However, no oral fluid HIV self‐test has yet been approved, reducing choice for Australian men. Uptake of HIV self‐testing has been limited; in surveys of the Sydney gay community during 2019 and 2020, fewer than 1% of HIV‐negative participants reported that their most recent HIV test had been a self‐test.^4^

Studies in Australia,^13^, ^14^ in other high income countries^15^, ^16^, ^17^ and in resource‐limited countries^18^, ^19^ have found high levels of usability and acceptability of HIV self‐tests among people at risk. To inform the further use of HIV self‐tests in Australia, we assessed the ease of use of HIV self‐test kits (oral fluid‐ or blood‐based), preferred HIV self‐test type, and difficulties encountered during HIV self‐testing by men who have sex with men.

## Methods

For our non‐blinded, randomised crossover trial, phase 2 of the Preferences and Usability of self‐test kits for HIV among gay and bisexual men (PUSH) study,^20^ we recruited participants who attended two publicly funded sexual health clinics in Melbourne and Sydney during 7 January – 10 December 2019. Men aged 18 years or more were eligible if they had ever previously had sex with a man, but were excluded if they reported living with HIV, could not speak or read English, could not provide informed consent, or could not meet all study requirements. Eligible participants were provided with an information sheet, and the research nurse explained the study procedures. Those who agreed to participate provided written consent. Our study complied with the 2010 CONSORT statement extension to randomised crossover trials.^21^

### Participant randomisation

Equal numbers of participants were randomised by an independent biostatistician to the two study arms using a computer‐generated sequence, stratified by site (maximum 200 participants from each site). Participants in one arm received the oral fluid‐based HIV self‐test first, those in the other arm the blood‐based HIV self‐test; in the second stage, each participant used the alternative self‐test. The test order for each participant was recorded in sequentially numbered sealed envelopes; each envelope was selected in order by the research nurse to maintain the pre‐generated random sequence, and was opened by the research nurse only after participant enrolment.

### HIV self‐test procedure

Participants were provided, without cost, two HIV self‐tests: an oral fluid‐based test (OraQuick Advance HIV1/2 rapid antibody test; not yet approved by the TGA) and a blood‐based test (Atomo HIV Self‐Test, Elion platform). The manufacturer of the oral fluid HIV test reports its sensitivity as 99.3% and specificity as 99.8%;^22^ the manufacturer of the blood HIV self‐test reports 99.6% sensitivity and 99.6% specificity.^23^

Participants could view manufacturers’ instructional videos for each kit on a tablet computer, and read the printed instructions included with the test kits; to reflect real‐world use, participants could choose freely whether and how to use each instruction type. Viewing the instructional video before purchasing the blood‐based kit was required in Australia during November 2018 – October 2021,^24^ but our study was designed before this requirement was introduced. During testing, undertaken in a private room in the clinic, participants were observed by a research nurse, but were not permitted to ask them questions about using the kits; they were instead referred to the provided instructions. All participants with reactive HIV self‐test results were offered confirmatory testing on the same day, and immediate supportive counselling by clinic staff.

### Data collection

Participants completed a multi‐part online questionnaire before testing (part 1: socio‐demographic characteristics, HIV and other sexually transmitted infection testing history, sexual behaviour) and after using each kit (ie, while waiting for the test result). In part 2 of the questionnaire, participants evaluated ease of use for each test step (five‐point Likert scales), generating an index (cumulative total) score ranging from 0 to 30 (six steps) for oral fluid HIV self‐testing and from 0 to 35 (seven steps) for blood HIV self‐testing. Finally, participants provided an overall rating of ease of use for each test (very difficult, somewhat difficult, slightly difficult, slightly easy, somewhat easy, very easy), and nominated the kit they preferred (Supporting Information, questionnaire).

During testing, the research nurse used a checklist to record whether participants exhibited any difficulty during each step of self‐testing. The checklist was based on manufacturer‐provided instructions for test use and a published checklist for observing HIV self‐testing.^25^

### Outcomes

The primary outcome was ease of performing HIV self‐testing, assessed as the proportion of participants who rated overall use as very easy, easy, or slightly easy (combined for our analyses as “easy to use”). Secondary outcomes were test preference (proportion of participants who preferred a particular HIV self‐test) and difficulties in self‐testing (proportions of participants for whom nurses recorded difficulties, by test step).

### Statistical analysis

Statistical analyses were performed in Stata 16. All participants originally allocated to a research arm were included in our analyses. A sample size of 167 participants was adequate to detect a 20 percentage point difference (60% *v* 40%) in the proportion of participants reporting that a HIV self‐test was easy to use with 80% power (α = 0.05) (*post hoc* power sample size calculation for simple 2 × 2 crossover design; assumed paired proportions with 10% correlation of repeated observations).

For the primary outcome, we assessed differences between the proportions of participants who described self‐testing as easy by estimating odds ratios [ORs] with 95% confidence intervals [CIs] in generalised mixed models with a binomial link function that accounted for within‐subject correlation associated with the crossover design. If the carryover effect was not statistically significant, we assessed the significance of differences in proportions reporting that the test was “easy to use” (binary) in McNemar tests. We repeated our analyses separately for men at high risk (anal sex without condoms during the preceding six months and not using PrEP) and those at usual risk, and for men frequently tested for HIV (at least every three months) and infrequent testers (groups based on questionnaire responses).

For the secondary outcomes, we estimated the proportions of men who preferred one or other HIV self‐test, with 95% CIs calculated using the exact method (binomial distribution). We summarise nurses’ observations of step‐specific difficulties as descriptive statistics.

In supplementary ordered logistic regression analyses, motivated by community interest in which groups of men might be interested in HIV self‐testing, we assessed the influence of demographic and behavioural characteristics reported to be associated with using HIV self‐testing, including age,^17^ ethnic background,^14^ sexual risk behaviour,^14^, ^15^, ^16^, ^17^ and HIV testing history,^26^ on the intentions of participants to use HIV self‐testing in the future, to offer an HIV self‐test kit to a regular partner, and to offer an HIV self‐test kit to a casual partner.

### Ethics approval

The South Eastern Sydney Local Health District – Northern Sector Hospital Human Research Ethics Committee (17/147), the Alfred Health Human Research Ethics Committee (486/17), the ACON Research Ethics Review Committee (2017/32), and the Thorne Harbour Health Community Research and Endorsement Panel (VAC/CREP/18/001) approved the study. We submitted a Clinical Trial Notification regarding the use of the OraQuick test to the TGA (CT‐2017‐CTN‐04321‐1; protocol: 17/147).

## Results

We recruited 170 eligible participants (Box 1); their median age was 34 years (interquartile range, 29–43 years), 144 identified as gay (85%), 96 were born outside Australia (57%), and 130 had university degrees (77%). A total of 125 participants (74%) had been tested for HIV during the preceding three months, and two participants had never been tested (Box 2).

Box 1 The acceptability and usability of two HIV self‐testing kits among Australian men who have sex with men: a randomised crossover trial. CONSORT flow chart*

**Figure:**
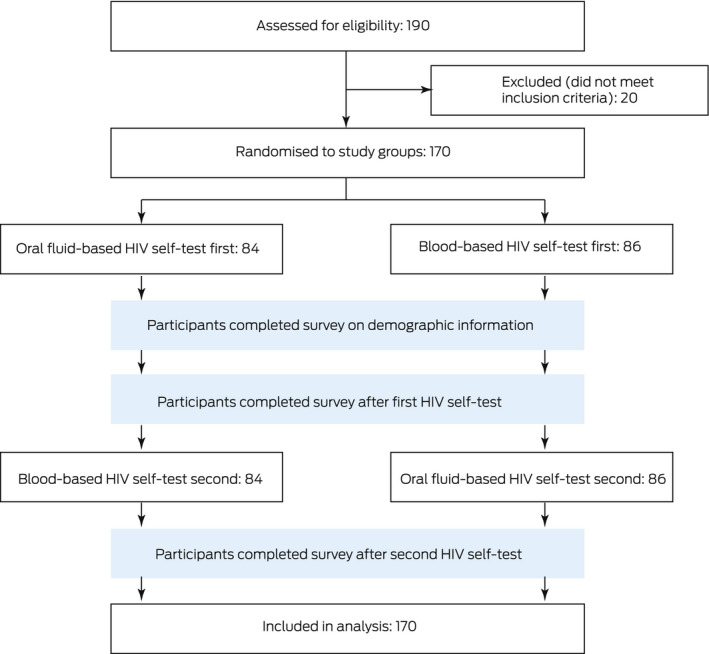
* All participants received the interventions as planned, and none were lost to follow‐up.

Box 2 **Socio‐demographic characteristics and sexual behaviours of the 170 study participants**

**Table:** 

Characteristic	Number
Sexuality	
Gay	144 (85%)
Bisexual	25 (15%)
Other	1 (1%)
Country of birth	
Australia	74 (44%)
Overseas	96 (57%)
Highest level of education	
Secondary school or less	14 (8%)
Trade certified/diploma/TAFE	26 (15%)
University degree	130 (77%)
Employed full time	
Yes	105 (62%)
No	65 (38%)
Male sexual partners in past six months	
Six or fewer	91 (54%)
More than six	79 (47%)
Sex with casual partners in past six months	
No casual partner	25 (15%)
Consistent condom use with casual partner	28 (19%)
Any condomless anal intercourse with casual partner	117 (81%)
Pre‐exposure prophylaxis in past six months	
Yes	101 (59%)
No	68 (40%)
Missing data	1 (1%)
Most recent HIV test*	
< 6 weeks	54 (32%)
6 weeks to 3 months	71 (42%)
3–12 months	30 (18%)
1–2 years ago	11 (6%)
More than 2 years or never tested	4 (2%)
Prior use of HIV self‐testing kits	
Yes	10 (6%)
No	155 (91%)
Missing data	5 (3%)

HIV = human immunodeficiency virus; TAFE = Technical and Further Education.

* Further details on to HIV testing attitudes and practice are included in the Supporting Information, table 1.

### Ease of use

Almost all participants (167 of 169 who responded to the question, 99%) reported finding the oral fluid HIV self‐test easy to use; 145 of 169 participants (86%) found the blood‐based HIV self‐test easy to use (Supporting Information, table 2). In generalised mixed models with a binomial link function, the oral fluid test was more likely than the blood test to be reported as being easy to use (OR, 3.0; 95% CI, 1.4–6.6), but a period effect was also evident (period 1 *v* 2: OR, 2.5; 95% CI: 1.2–5.2); sequence and carryover effects were not statistically significant. Men at lower risk (OR, 3.0; 95% CI, 1.2–7.4) and those regularly tested for HIV (OR, 2.8; 95% CI, 1.2–6.8) were more likely to report the oral fluid test easy to use than the blood test, but not men at high risk or those less frequently tested (Box 3).

Box 3 **Ease of use of HIV self‐test kits by men who have sex with men: generalised mixed models with a binomial link function**

**Table:** 

Effect	OR (95% CI)	P	P^*^
All participants (*N* = 170)			
Main effect (oral fluid *v* blood test)	3.0 (1.4–6.6)	0.006	0.019
Period effect (period 1 *v* 2)	2.5 (1.2–5.3)	0.018	
Sequence effect (oral fluid first *v* blood first)	0.6 (0.3–1.2)	0.12	
Carryover effect	0.5 (0.1–1.9)	0.30	
High risk participants (*N* = 49)^†^			
Main effect (oral fluid *v* blood test)	3.3 (0.6–19)	0.18	0.48
Period effect (period 1 *v* 2)	1.5 (0.4–6.2)	0.58	
Sequence effect (oral fluid first *v* blood first)	0.8 (0.2–3.1)	0.73	
Carryover effect	0.7 (0.1–15)	0.84	
Low risk participants (*N* = 121)			
Main effect (oral fluid *v* blood test)	3.3 (0.6–19)	0.18	0.024
Period effect (period 1 *v* 2)	1.5 (0.4–6.2)	0.58	
Sequence effect (oral fluid first *v* blood first)	0.8 (0.2–3.1)	0.73	
Carryover effect	0.7 (0.1–15)	0.84	
Frequent testers (*N* = 129)^‡^			
Main effect (oral fluid *v* blood test)	2.8 (1.2–6.8)	0.023	0.11
Period effect (period 1 *v* 2)	1.6 (0.9–2.8)	0.12	
Sequence effect (oral fluid first *v* blood first)	0.6 (0.3–1.1)	0.11	
Carryover effect	0.4 (0.1–1.6)	0.18	
Infrequent testers (*N* = 39)			
Main effect (oral fluid *v* blood test)	4.0 (0.7–23)	0.12	0.06
Period effect (period 1 *v* 2)	2.5 (0.5–12)	0.25	
Sequence effect (oral fluid first *v* blood first)	0.8 (0.2–3.0)	0.75	
Carryover effect	NA	—	

CI = confidence interval; HIV = human immunodeficiency virus; NA = not applicable (model did not converge); OR = odds ratio.

* McNemar tests for difference between tests in proportions of participants reporting “easy to use”.

† Men who had anal sex without condoms during the preceding six months and were not using pre‐exposure prophylaxis.

‡ Tested at least once every three months.

The most difficult step according to participant evaluations was placing the oral fluid HIV test tube in the stand (43 of 169 participants [25%] rated it as very easy, easy or slightly easy) and filling the collection channel with enough blood for the blood‐based HIV self‐test (58 of 166 [35%]). Most oral fluid‐ (158 of 169, 94%) and blood‐based self‐test users (159 of 169, 94%) used only the written instructions; ten of 169 oral fluid (6%) and eight of 169 blood self‐test users (5%) viewed the video instructions (Supporting Information, table 2).

### Preferred test

The oral fluid HIV self‐test was preferred by 98 of 170 participants (58%; 95% CI, 50–65%), the blood self‐test by 69 participants (41%; 95% CI, 33–48%); three participants (2%) had no preference. Features favouring the oral fluid test were ease of use (55 of 170, 32%), no pain (38 of 170, 22%), and not requiring a blood specimen (35 of 170, 21%). Features favouring the blood‐based HIV self‐test included greater accuracy (49 of 170, 29%), ease of setting up the test kit and testing (16 of 170, 9%), and easier‐to‐understand instructions (ten of 170, 6%) (Supporting Information, table 3).

### Nurses’ observations of self‐testing

The final step in the HIV self‐test process was completed by 150 of 151 participants using the oral fluid HIV self‐test and 138 of 155 participants using the blood‐based self‐test. According to the research nurses, the proportions of oral fluid self‐test users who had difficulties with self‐testing steps were largest for placing the buffer solution into the stand (40 of 170, 24%), swabbing both the upper and lower gums (23 of 169, 14%) and reading the result within 20–40 minutes (17 of 151, 11%). For blood self‐test users, the proportions were largest for filling the device test channel (69 of 170, 41%), squeezing their finger firmly enough to generate a blood drop (42 of 170, 25%), massaging their finger sufficiently before lancing (26 of 169, 15%), and reading test results within 15–20 minutes (16 of 155, 10%). Eighty‐three of 160 oral fluid HIV self‐test users (52%) and 62 of 165 blood HIV self‐test users (38%) did not check the kit expiry date (Box 4; Supporting Information, table 4).

Box 4 Self‐testing steps completed by participants, by HIV self‐test kit type*

**Figure:**
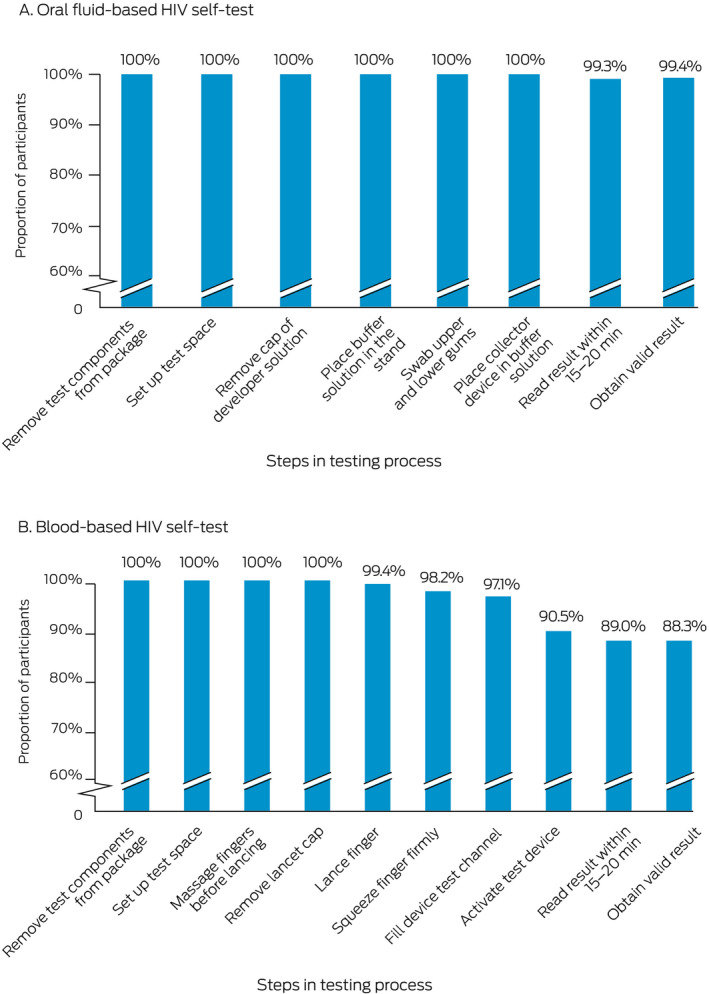
* The denominator for each step is the number of participants who attempted the step; the numerator is the number who completed the step. The underlying data for these graphs is provided in the Supporting Information, table 4.

Of those who completed the tests, no participant received an invalid oral fluid HIV self‐test result; two of 162 participants (1%) received invalid blood HIV self‐test results (ie, could not obtain an interpretable result despite completing the test steps).

### Future use of HIV self‐testing

Of 166 respondents to questionnaire question 47, 147 were likely to use self‐testing again (89%), 129 to offer it to a regular partner (78%), and 111 to offer it to a casual partner (67%). After adjusting for age, education level, and ethnic background, characteristics associated with higher odds of using HIV self‐testing in the future were overseas birth (adjusted OR [aOR], 3.07; 95% CI, 1.42–6.64), self‐evaluated ease of use (oral fluid: aOR, 1.14; 95% CI, 1.06–1.24; blood: aOR, 1.25; 95% CI, 1.13–1.38), and self‐evaluated confidence in use (oral fluid: aOR, 1.11; 95% CI, 1.03–1.20; blood: aOR, 1.17; 95% CI, 1.06–1.29) (Supporting Information, table 5). In the univariate analysis, PrEP use was associated with lower odds of self‐testing in the future (OR, 0.54; 95% CI, 0.30–0.97), but this was not statistically significant in the multivariable analysis (aOR, 0.55; 95% CI, 0.30–1.00).

Characteristics associated with higher odds of providing HIV self‐test kits to regular or casual partners included ease and confidence with using the kits, overseas birth; for offering a self‐test kit to a casual partner, identifying as gay and having more than six male partners in the past six months were also factors (Supporting Information, table 6).

## Discussion

We found Australian men who have sex with men regarded two HIV self‐tests as easy to use and acceptable. The likelihood of being regarded as easy to use was three times as high for the oral fluid test as for the blood self‐test, and a larger proportion of participants preferred the oral fluid test (58% *v* 41% for the blood‐based test). No participants received invalid results with the oral fluid‐based HIV self‐test, and only 1% did so with the blood‐based test.

Despite these encouraging findings, uptake of HIV self‐testing has been low in Australia,^4^, ^5^ probably because of limited test choice and availability, lack of advertising, and cost.^27^ Only one blood‐based HIV self‐test has been approved by the TGA, and no oral fluid‐based test. As men preferred one or other test type for different reasons — ease of use for the oral fluid test, perceived accuracy for the blood test — it would be helpful if both kit types were available. Until October 2021, the blood‐based HIV self‐test could only be bought online, as the TGA required that an instructional video be viewed prior to purchase. Online access makes HIV testing more available, particularly to people living in rural and remote areas. However, access to HIV self‐test kits from a range of locations, as is the case overseas, is preferable; informed by the findings from our study, the requirement to watch the video has been lifted,^28^ and the blood‐based kits are now available from pharmacies in Australia. Nine of ten participants in our sample completed all steps without the video, and 98.8% received valid test results.

Most men preferred written to video instructions, indicating the need for printed instructions to be clear and to explain the steps that users found most difficult. For example, instructions should emphasise the need to set a timer and to check the kit expiry date, which could be printed more prominently. The blood‐based test kit instructions recommended checking the expiry date, and a larger proportion of its users indeed checked the date (62% *v* 48%). An earlier Australian study using the same blood‐based HIV self‐test kit similarly found that 45% of participants checked its expiry date.^13^

Participants born overseas were three times as likely to want to use self‐testing again as those born in Australia. Given the high late diagnosis rate for overseas‐born men who have sex with men, HIV self‐testing, coupled with other culturally appropriate approaches, should be a greater focus in our HIV prevention strategy. As many overseas‐born men are ineligible for subsidised health care (Medicare) and therefore subsidised HIV testing, a targeted self‐testing program should consider reducing its cost.

### Limitations

First, participants recruited from sexual health centres may have relatively high levels of health education and motivation, and therefore not be representative of all men who have sex with men. Second, HIV self‐test kits provided instructions in English only, for which reason we exclusively recruited participants who could speak and read English. Third, the presence of a nurse during self‐testing may have resulted in participants being more careful during the test. Further, three different nurses were involved in the study, potentially introducing measurement bias, although we mitigated the risk by ensuring the checklist items were as objective as possible.

### Conclusion

The oral fluid‐based HIV self‐test was regarded by participants as easier to use than the blood‐based self‐test, and was preferred by a larger proportion of men, but the usability and acceptability of both kits were high. Despite the very low proportion of invalid test results, further refinements to make HIV self‐testing easier and more reliable are important.

## Open access

Open access publishing facilitated by Monash University, as part of the Wiley ‐ Monash University agreement via the Council of Australian University Librarians.

## Competing interests

No relevant disclosures.

## Supporting information


Appendix
