# Supplementary material for: The acceptability and usability of two HIV self‐test kits among men who have sex with men: a randomised crossover trial
Source: Med J Aust. 2022 Jul 12;217(3):149–54. doi: 10.5694/mja2.51641 (PMC9542976; doi:10.5694/mja2.51641)
Supplement: Supplementary file 1 — Appendix [file MJA2-217-149-s001.pdf]

The Medical Journal of Australia

# Supporting Information

## Supplementary methods and results

**This appendix was part of the submitted manuscript and has been peer reviewed.**
**It is posted as supplied by the authors.**

Appendix to: Lee DYL, Ong JJ, Smith K, et al. The acceptability and usability of two HIV self-test kits among men who have sex with men: a randomised crossover trial. *Med J Aust* 2022; doi: 10.5694/mja2.51641.

**Survey: Preferences and usability of self-test kits for HIV among gay and bisexual men**

Nurse to enter participant study ID before handing the survey device to participant. This will be needed to link the questionnaire to patient consent form and the order of self-testing kits.

Please enter participant's **study ID**: __________

**<u>Information for participants</u>**

You are invited to complete this questionnaire because you have agreed to participate in this study. All the information you provide will be treated as strictly confidential. You will not be identified, and we will only use a unique number to keep track of your data. The entire questionnaire and self-testing will take about 45 minutes to complete.

The study nurse can answer any questions you may have about study procedures but will not be able to provide assistance with self-testing.

## Section 1: About you

1. How old are you? ____ years
2. What sex were you assigned at birth? (i.e. sex specified on your original birth certificate)
    a. Male
    b. Female
3. Which of the following best describes your current gender identity?
    a. Male
    b. Female
    c. Transman
    d. Transwoman
    e. Indigenous Brotherboy
    f. Indigenous Sistergirl
    g. Non-binary/gender fluid
    h. Different identity: please specify ______________
4. Were you born with a variation of sex characteristics (this is sometimes called 'intersex')?
    a. Yes
    b. No
    c. Prefer not to answer
5. Have you ever had sex with another man?
    a. Yes
    b. No
6. Do you think of yourself as:
    - ☐ Gay / Homosexual
    - ☐ Bisexual
    - ☐ Other, please specify ____________
7. Are you of Aboriginal or Torres Strait Islander origin?
    - Yes, Aboriginal only
    - Yes, Torres Strait Islander only
    - Yes, both Aboriginal and Torres Strait Islander
8. What is your ethnic background (e.g. Dutch, Greek, Vietnamese, Lebanese)?
    - ☐ Anglo-Australian
    - ☐ Other (please specify) __________
9. Where were you born?
    - ☐ Australia (Go to Q11)
    - ☐ Overseas, [drop down list of countries/regions will be provided]
10. In what year did you first come to live in Australia? __________
11. What is your postcode of residence? ☐ ☐ ☐ ☐
12. What is the highest level of education you have completed?
    - ☐ Primary school
    - ☐ Up to Year 10
    - ☐ Year 12 / HSC or equivalent
    - ☐ Trade certified / diploma / TAFE
    - ☐ Undergraduate university
    - ☐ Postgraduate university
13. What is your current employment status?
    (Tick as many as apply)
    - ☐ Employed full-time
    - ☐ Employed part-time
    - ☐ On pension / social security
    - ☐ Student
    - ☐ Unemployed
    - ☐ Other, please specify __________

**14.** How many of your friends are gay or homosexual men?
- ☐ None
- ☐ A few
- ☐ Some
- ☐ Most
- ☐ All

**15.** How much of your free time is spent with gay or homosexual men?
- ☐ None
- ☐ A little
- ☐ Some
- ☐ A lot

## Section 2: Sex in the last 6 months

**16.** In the **last 6 months**, how many different men have you had sex with? _______ men

**17.** In the **last 6 months**, how often have you had group sex involving at least two other men?
- ☐ Every week
- ☐ Monthly
- ☐ A few times, but no more than 3-5 times
- ☐ Once
- ☐ Never

## Section 3: Sex with Regular Male Partner(s)

**18.** In the **last 6 months**, how many **REGULAR** (boyfriends/fuck buddies/lovers) partner(s) have you had anal sex with?

________ regular partners
- ☐ None (Go to Q22)

**19.** In the **last 6 months**, how often did you use condoms for anal sex with your **REGULAR** male partner(s)?
- ☐ Always
- ☐ Often
- ☐ Occasionally
- ☐ Never

Below: If you have more than one regular male partner, answer the next question in relation to your **MAIN** or **PRIMARY** regular partner. If you cannot choose a main or primary partner, then consider the one regular partner you have been with the longest period of time.

**20.** What was the result of your REGULAR male partner's **last** HIV test?
- ☐ Positive
- ☐ Negative (go to Q22)
- ☐ I don't know / he hasn't had a test (go to Q22)

**21.** Is he on treatment and has an undetectable viral load?
- ☐ Yes
- ☐ No
- ☐ Don't know

## Section 4: Sex with Casual Partner(s)

**22.** In the **last 6 months**, how many **CASUAL** male partner(s) have you had anal sex with?
_____________ casual partners
☐ None (Go to 26)

**23.** In the **last 6 months**, how often did you use condoms for anal sex with your **CASUAL** male partner(s)?
a. Always
b. Often
c. Occasionally
d. Never

**24.** In the **last 6 months**, how many of your casual male partners did you tell **your** HIV status before sex?
☐ None
☐ Some
☐ All

**25.** In the **last 6 months**, how many of your casual male partners told you **their** HIV status before sex?
☐ None
☐ Some
☐ All

**26.** In the **last 6 months**, did you take anti-HIV medication regularly to protect yourself from HIV – i.e. pre-exposure prophylaxis (**PrEP**)?
☐ I've never heard about PrEP
☐ No
☐ Yes, I was prescribed anti-HIV medication to take every day
☐ Yes, I took anti-HIV medication that was not prescribed

## Section 5: HIV / STI testing

**27.** Have you ever had an HIV test?
- ☐ Yes
- ☐ No (Part 1 Complete)

**28.** How often do you usually have an HIV test?
- ☐ Monthly
- ☐ Every three months
- ☐ Every six months
- ☐ Annually
- ☐ Less than once a year

**29.** When were you last tested for HIV?
- ☐ Within the last 6 weeks
- ☐ 6 weeks to 3 months ago
- ☐ 3 months to 1 year ago
- ☐ 1 - 2 years ago
- ☐ More than 2 years ago

**30.** Where did you have your last HIV test?
- ☐ GP
- ☐ Sexual health clinic
- ☐ Hospital
- ☐ Private home
- ☐ Community-based service e.g. a[TEST] or Pronto!
- ☐ Somewhere else, please specify ___________

**31.** Why did you have your **last** HIV test?
(Tick as many as apply)
- ☐ I wanted to know my status
- ☐ I did something risky
- ☐ My partner did something risky
- ☐ I had a symptom or an illness that made me worry
- ☐ It was part of my regular testing pattern
- ☐ It was part of screening/follow-up testing for pre-exposure prophylaxis (PrEP)
- ☐ I changed partners
- ☐ My partner asked me to
- ☐ I had sex with someone I knew or thought was HIV positive
- ☐ My doctor suggested it
- ☐ An agreement with my partner not to use condoms in our relationship
- ☐ Other, please specify ____________________________

**32.** When you go to your doctor for an HIV test, how much do you feel:

| | Not at all | A little | Somewhat | Very much |
|---|---|---|---|---|
| Embarrassed when you have to discuss your sex life | ☐ | ☐ | ☐ | ☐ |
| Embarrassed when asking for an HIV test | ☐ | ☐ | ☐ | ☐ |
| Anxious when waiting for results | ☐ | ☐ | ☐ | ☐ |

**33.** Over the **past year**, have you ever put off or decided against having an HIV test?
□ Yes
□ No (skip to Qu 35)

**34.** Why did you put off or decide against having an HIV test?:
(Tick as many as apply)
- ☐ I would prefer to test myself using a self-test but none are available
- ☐ I haven't done anything risky
- ☐ I don't want to know my status
- ☐ I don't want to be seen getting a sexual health check-up
- ☐ I don't want my family or other people to know
- ☐ The process of getting tested is too much hassle
- ☐ I don't like having to return for the results
- ☐ I don't want to go to a clinic or doctor to get tested
- ☐ I don't want to have to discuss my sex life
- ☐ I don't like having blood taken for the test
- ☐ I haven't had any symptoms or an illness that made me worry
- ☐ Other, please specify ________________________________

**35. Have you ever tested yourself for HIV using an HIV self-test kit?**
□ Yes
□ No

**36. How have you previously obtained HIV self-test kit(s)?**
□ I participated in the FORTH study (HIV self-testing trial)
□ Purchased online
□ Brought from overseas
□ Given to me by someone
□ Other (please specify):

**37.** [If yes] What type of HIV self-test have you used? (tick any that apply)
□ Oral fluid self-ytest
□ Fingerprick (blood) self-test

**PART-1 IS NOW COMPLETE. PLEASE USE THE SELF-TEST PROVIDED TO YOU TO TEST YOURSELF AND THEN PROCEED TO NEXT PART, OR ASK THE NURSE FOR ASSISTANCE IF UNSURE.**

## QUESTIONNAIRE PART-2A

Please complete this part after you have used the self-test kit provided to you. This part asks about your experience using that test, and interpretation of your test result.

What was the first test you used to test yourself? :
□ OraQuick HIV Self-Test (oral test)
□ Atomo HIV Self Test (fingerprick test)

**38.** Thinking about your test, how did you find performing each of the steps below?
[options presented below will depend on which test the participant had used]

| **OraQuick HIV Self- Test** | **Very difficult** | **Difficult** | **Slightly difficult** | **Slightly easy** | **Easy** | **Very easy** |
|---|---|---|---|---|---|---|
| Removing the cap from the tube | □ | □ | □ | □ | □ | □ |
| Setting tube in stand | □ | □ | □ | □ | □ | □ |
| Swabbing upper and lower gums | □ | □ | □ | □ | □ | □ |
| Inserting test device in tube | □ | □ | □ | □ | □ | □ |
| Timing the test | □ | □ | □ | □ | □ | □ |
| Interpreting the results | □ | □ | □ | □ | □ | □ |

| **Atomo HIV Self test** | **Very difficult** | **Difficult** | **Slightly difficult** | **Slightly easy** | **Easy** | **Very easy** |
|---|---|---|---|---|---|---|
| Removing green safety cap | □ | □ | □ | □ | □ | □ |
| Massaging the finger firmly to stimulate blood flow | □ | □ | □ | □ | □ | □ |
| Pricking finger | □ | □ | □ | □ | □ | □ |
| Filling channel with blood | □ | □ | □ | □ | □ | □ |
| Activating the test by pressing the activate test button | □ | □ | □ | □ | □ | □ |
| Timing the test | □ | □ | □ | □ | □ | □ |
| Interpreting the results | □ | □ | □ | □ | □ | □ |

**39.** Thinking about your test, how confident are you that you performed the steps below correctly?
[options presented below will depend on which test the participant had used]

| **OraQuick HIV Self Test** | **Not at all confident** | **Not very confident** | **Confident** | **Very confident** | **Completely confident** |
|---|---|---|---|---|---|
| Removing the cap from the tube | ☐ | ☐ | ☐ | ☐ | ☐ |
| Setting tube in stand | ☐ | ☐ | ☐ | ☐ | ☐ |
| Swabbing upper and lower gums | ☐ | ☐ | ☐ | ☐ | ☐ |
| Inserting test device in tube | ☐ | ☐ | ☐ | ☐ | ☐ |
| Timing the test | ☐ | ☐ | ☐ | ☐ | ☐ |
| Interpreting the results | ☐ | ☐ | ☐ | ☐ | ☐ |

| **Atomo HIV Self test** | **Not at all confident** | **Not very confident** | **Confident** | **Very confident** | **Completely confident** |
|---|---|---|---|---|---|
| Removing green safety cap | ☐ | ☐ | ☐ | ☐ | ☐ |
| Massaging the finger firmly to stimulate blood flow | ☐ | ☐ | ☐ | ☐ | ☐ |
| Pricking the finger | ☐ | ☐ | ☐ | ☐ | ☐ |
| Filling channel with blood | ☐ | ☐ | ☐ | ☐ | ☐ |
| Activating the test by pressing the activate test button | ☐ | ☐ | ☐ | ☐ | ☐ |
| Timing the test | ☐ | ☐ | ☐ | ☐ | ☐ |
| Interpreting the results | ☐ | ☐ | ☐ | ☐ | ☐ |

**40.** Thinking about your first test, which of the instructions below did you use?
(Tick as many as apply)

☐ Instructions for use included in test kit

☐ How-to-Video

**41.** How helpful did you find the instructions?

| | Very unhelpful | Unhelpful | Slightly unhelpful | Slightly helpful | Helpful | Very helpful |
|---|---|---|---|---|---|---|
| Kit instructions | ☐ | ☐ | ☐ | ☐ | ☐ | ☐ |
| How-to-Video | ☐ | ☐ | ☐ | ☐ | ☐ | ☐ |

**42.** How easy was it for you to understand the instructions?

| | Very difficult | Difficult | Slightly difficult | Slightly easy | Easy | Very easy |
|---|---|---|---|---|---|---|
| Kit instructions | ☐ | ☐ | ☐ | ☐ | ☐ | ☐ |
| How-to-Video | ☐ | ☐ | ☐ | ☐ | ☐ | ☐ |

**43.** Thinking about your first test, how easy was it for you to test yourself overall using that test?

- ☐ Very difficult
- ☐ Somewhat difficult
- ☐ Slightly difficult
- ☐ Slightly easy
- ☐ Somewhat easy
- ☐ Very easy

**NOW USE THE SECOND OF TWO SELF-TESTS PROVIDED TO YOU TO TEST YOURSELF AND THEN PROCEED TO NEXT PART, OR ASK THE NURSE FOR ASSISTANCE IF UNSURE.**

**QUESTIONNAIRE PART-2B**

Please complete this part after you have used the second of two self-tests provided to you. This part asks about your experience using that test, and interpretation of your test result.

What was the second test you used to test yourself?:
□ OraQuick HIV Self-Test (oral test)
□ Atomo HIV Self Test (fingerprick test)

REPEAT ABOVE QUESTIONS FOR TEST 2

**44.** Which of the two tests did you prefer?:
☐ OraQuick HIV Self-Test (oral test)
☐ Atomo HIV Self Test (fingerprick test)

**45.** Why do you prefer this test? Please specify:

**QUESTIONNAIRE PART-C**

This part of the questionnaire asks about your preferences for availability and use of HIV self-tests in the future.

**46.** If available in the future, how likely it is that you would purchase HIV self-tests from the sources below?

| | **Very unlikely** | **Unlikely** | **Slightly unlikely** | **Slightly likely** | **Likely** | **Very likely** |
|---|---|---|---|---|---|---|
| Online / internet | ☐ | ☐ | ☐ | ☐ | ☐ | ☐ |
| A vending machine | ☐ | ☐ | ☐ | ☐ | ☐ | ☐ |
| Sexual Health Centre | ☐ | ☐ | ☐ | ☐ | ☐ | ☐ |
| General Practice | ☐ | ☐ | ☐ | ☐ | ☐ | ☐ |
| Community pharmacy/ chemist | ☐ | ☐ | ☐ | ☐ | ☐ | ☐ |
| A community organisation (e.g. ACON, VAC) | ☐ | ☐ | ☐ | ☐ | ☐ | ☐ |
| Sex on premises venue (sauna or sex club) | ☐ | ☐ | ☐ | ☐ | ☐ | ☐ |
| Grocery/ convenience store | ☐ | ☐ | ☐ | ☐ | ☐ | ☐ |

**47.** If available, how likely is it that you would use HIV self-tests to:

| | **Very unlikely** | **Unlikely** | **Slightly unlikely** | **Slightly likely** | **Likely** | **Very likely** |
|---|---|---|---|---|---|---|
| Test yourself | ☐ | ☐ | ☐ | ☐ | ☐ | ☐ |
| Offer the test to a regular partner (e.g. boyfriend/lover) | ☐ | ☐ | ☐ | ☐ | ☐ | ☐ |
| Offer the test to a fuckbuddy | ☐ | ☐ | ☐ | ☐ | ☐ | ☐ |
| Offer the test to a casual partner | ☐ | ☐ | ☐ | ☐ | ☐ | ☐ |

**48.** If you got a REACTIVE (i.e. positive) HIV self-test result, how likely is it that you would go to a doctor or clinic for further confirmatory testing?

- ☐ Very unlikely
- ☐ Somewhat unlikely
- ☐ Slightly unlikely
- ☐ Slightly likely
- ☐ Somewhat likely
- ☐ Very likely

**49.** What is the maximum you would be willing to pay for a HIV self-test?
AUD ______

**50.** Based on your experience using HIV self-tests today, how likely is it that you would recommend self-tests to other men?

- ☐ Very unlikely
- ☐ Somewhat unlikely
- ☐ Slightly unlikely
- ☐ Slightly likely
- ☐ Somewhat likely
- ☐ Very likely

---

**End of survey: Please inform the nurse that you have completed all requirements of the study. Thank you for your valuable contribution to this important research!**

**Table 1. Attitudes to HIV testing among Australian gay, bisexual and other men who have sex with men [**

| Characteristic | n (%) |
|---|---|
| **Last HIV test (N=170)** | |
| <6 weeks ago | 54 (32%) |
| 6 weeks - 3 months ago | 71 (42%) |
| 3 months - 1 year ago | 30 (18%) |
| 1-2 years ago | 11 (7%) |
| >2 years ago | 2 (1%) |
| Never been tested | 2 (1%) |
| **Reasons for last HIV test (N=168)** | |
| Part of regular testing pattern | 109 (65%) |
| Desire to know status | 102 (61%) |
| Part of PrEP screening/follow-up testing | 60 (36%) |
| Risky sexual behaviour (own or partner's) | 37 (22%) |
| Change of partners | 14 (8%) |
| Symptom or illness that caused concern | 7 (4%) |
| Other | 16 (10%) |
| **Ever put off a HIV test in the last 12 months (N=170)** | |
| Yes | 13 (8%) |
| No | 157 (92%) |
| **Reasons for putting off HIV test (N=13)** | |
| Hassle of testing process (incl. getting tested, returning for results) | 10 (77%) |
| No risky sexual behaviours | 7 (54%) |
| No symptoms/illnesses that made them worry | 6 (46%) |
| Not wanting to go to a clinic or doctor | 5 (38%) |
| Preference for a self-test at home | 4 (31%) |
| Not wanting status to be known (either to self or others) | 3 (23%) |
| Not wanting to be seen getting a sexual health check-up | 3 (23%) |
| Not wanting to discuss sex life | 1 (8%) |
| Other | 1 (8%) |
| **Level of embarrassment experienced when asking for a HIV test when going for a HIV test (N=168)** | |
| Not at all | 110 (65%) |
| A little | 42 (25%) |
| Somewhat | 11 (7%) |
| Very much | 3 (2%) |
| Missing data | 2 (1%) |
| **Level of embarrassment experienced when needing to discuss sex life when going for a HIV test (N=168)** | |
| Not at all | 143 (85%) |
| A little | 15 (9%) |
| Somewhat | 4 (2%) |
| Very much | 4 (2%) |
| Missing data | 2 (1%) |
| **Level of anxiety experienced when waiting for results from a HIV test (N=168)** | |
| Not at all | 66 (39%) |
| A little | 70 (41%) |
| Somewhat | 20 (12%) |
| Very much | 11 (7%) |
| Missing data | 2 (1%) |

**Table 2. Experience of HIV self-testing among Australian men who have sex with men***

| | n (%) |
|---|---|
| **First test administered (N=170)** | |
| Oral HIV self-test | 84 (49%) |
| Blood-based HIV self-test | 86 (51%) |
| **Participants who evaluated performing the following oral fluid HIV self-testing steps as being very easy, easy or slightly easy** | |
| Removing the cap from the tube (N=169) | 156 (92%) |
| Placing the test tube in the stand (N=169) | 126 (75%) |
| Swabbing upper and lower gums (N=169) | 164 (97%) |
| Inserting test device into tube(N=169) | 165 (98%) |
| Timing the test (N=169) | 166 (98%) |
| Reading test results (N=168) | 164 (98%) |
| Interpreting test results (N=169) | 162 (96%) |
| **Participants who evaluated performing the following blood HIV self-testing steps as being very easy, easy or slightly easy** | |
| Removing green safety cap (N=169) | 159 (94%) |
| Massaging finger firmly to stimulate blood flow (N=169) | 151 (89%) |
| Pricking finger with the lancet (N=168) | 140 (83%) |
| Filling collection channel with blood (N=166) | 108 (65%) |
| Activating the test by pressing the activate test button (N=160) | 146 (91%) |
| Timing the test (N=160) | 154 (96%) |
| Reading the test results (N=158) | 151 (96%) |
| Interpreting the test results (N=158) | 148 (94%) |
| **Participants who evaluated their confidence in performing the following oral fluid HIV self-testing steps as being somewhat, very or completely confident** | |
| Removing the cap from the tube (N=169) | 167 (99%) |
| Placing the test tube into the stand (N=169) | 155 (92%) |
| Swabbing upper and lower gums (N=169) | 164 (97%) |
| Inserting the test device into the tube (N=169) | 168 (99%) |
| Timing the test (N=169) | 168 (99%) |
| Reading test results (N=168) | 165 (98%) |
| Interpreting test results (N=168) | 165 (98%) |
| **Participants who evaluated their confidence in performing the following blood-based HIV self-testing steps as being somewhat, very or completely confident** | |
| Removing green safety cap (N=167) | 159 (95%) |
| Massaging finger firmly to stimulate blood flow (N=168) | 160 (95%) |
| Pricking finger with the lancet (N=167) | 154 (92%) |
| Filling collection channel with blood (N=165) | 138 (84%) |
| Activating the test by pressing the activate test button (N=159) | 151 (95%) |
| Timing the test (N=158) | 155 (98%) |
| Reading the test results (N=158) | 154 (97%) |
| Interpreting the test results (N=159) | 153 (96%) |
| **Instructions used to help with the oral HIV self-testing process (N=169)** | |
| Written instructions only | 158 (93%) |
| Video instructions only | 1 (1%) |
| Both | 9 (5%) |
| Missing data | 1 (1%) |
| **Instructions used to help with the blood-based HIV self-testing process (N=169)** | |
| Written instructions only | 159 (94%) |
| Video instructions only | 1 (1%) |
| Both | 7 (4%) |
| Missing data | 2 (1%) |
| **Participants who found written instructions slightly helpful, helpful or very helpful** | |
| Oral HIV self-test (N=167) | 159 (95%) |
| Blood-based HIV self-test (N=166) | 158 (95%) |
| **Participants who found overall ease of testing very easy, easy or slightly easy (N=169)** | |
| Oral HIV self-test | 167 (99%) |
| Blood-based HIV self-test | 145 (86%) |

**Table 3. Experience of HIV self-testing among Australian gay, bisexual and other men who have sex with men***

| | |
|---|---|
| **Preferred method of self-testing (N=170)** | |
| Oral HIV self-testing | 98 (58%) |
| Blood-based HIV self-testing | 69 (41%) |
| No preference | 3 (2%) |
| **Reasons for preferring oral fluid HIV self-testing (N=170)** | |
| Easy to use | 55 (32%) |
| No pain/prick | 38 (22%) |
| No blood | 35 (21%) |
| Less invasive | 10 (6%) |
| Not messy/clean process | 6 (4%) |
| Faster process | 5 (3%) |
| Longer window to check results | 1 (1%) |
| **Reasons for preferring blood-based HIV self-testing (N=170)** | |
| Perceived to be more accurate/trustworthy/efficacious | 49 (29%) |
| Equipment easier to set up and use | 16 (9%) |
| Instructions easier to understand | 10 (6%) |
| Quicker process | 8 (5%) |
| Not confident about swabbing gums | 4 (2%) |
| More familiar process | 3 (2%) |
| **Participants reporting likelihood of being slightly likely, likely or very likely to use HIV self-testing in the future (N=166)** | |
| To test self | 147 (89%) |
| To offer regular partner | 129 (78%) |
| To offer casual partner | 111 (67%) |

**Table 4. Nurses' observations of participant difficulties**

| | n (%) |
|---|---|
| **Patients observed to have difficulties with the following steps (oral HIV self-testing )** | |
| Removing test components from package (N=169) | 11 (7%) |
| Setting up test space (N=169) | 6 (4%) |
| Removing cap of developer solution (N=170) | 5 (3%) |
| Placing buffer solution in the stand (N=170) | 40 (24%) |
| Swabbing upper and lower gums (N=169) | 23 (14%) |
| Placing collector device in buffer solution (N=170) | 7 (4%) |
| Reading result in 20-40mins (N=151) | 17 (11%) |
| **Patients observed to have omitted or failed to perform correctly the following steps (oral HIV self-testing )** | |
| Checking expiry date (N=160) | 83 (52%) |
| Disposing of test kit (N=158) | 51 (32%) |
| **Patients observed to have difficulties with the following steps (blood-based HIV self-testing )** | |
| Removing test components from package (N=169) | 8 (5%) |
| Setting up test space (N=169) | 3 (2%) |
| Massaging finger before lancing (N=169) | 26 (15%) |
| Removing lancet cap (N=170) | 10 (6%) |
| Lancing finger (N=169) | 15 (9%) |
| Squeezing finger firmly (N=170) | 42 (25%) |
| Filling device test channel (N=170) | 69 (41%) |
| Activating test device (N=169) | 7 (4%) |
| Reading result within 15-20mins (N=155) | 16 (10%) |
| **Patients observed to have omitted or failed to perform correctly the following steps (blood-based HIV self-testing )** | |
| Checking expiry date (N=165) | 62 (38%) |
| Cleaning hands before test (N=167) | 24 (14%) |
| Disposing of test kit (N=162) | 48 (30%) |
| **Number of participants who completed each step of the oral HIV self-testing process** | |
| Removing test components from package (N=169) | 169 (100%) |
| Setting up test space (N=169) | 169 (100%) |
| Removing cap of developer solution (N=170) | 170 (100%) |
| Placing buffer solution in the stand (N=170) | 170 (100%) |
| Swabbing upper and lower gums (N=169) | 169 (100%) |
| Placing collector device in buffer solution (N=170) | 170 (100%) |
| Reading result in 20-40mins (N=151) | 150 (99%) |
| Obtained valid result (N=162) | 161 (99%) |
| **Number of participants who completed each step of the blood-based HIV self-testing process** | |
| Removing test components from package (N=169) | 169 (100%) |
| Setting up test space (N=169) | 169 (100%) |
| Massaging finger before lancing (N=169) | 169 (100%) |
| Removing lancet cap (N=170) | 170 (100%) |
| Lancing finger (N=169) | 168 (99%) |
| Squeezing finger firmly (N=170) | 167 (98%) |
| Filling device test channel (N=170) | 165 (97%) |
| Activating test device (N=169) | 153 (90%) |
| Reading result within 15-20mins (N=155) | 138 (89%) |
| Obtained valid result (N=162) | 153 (88%) |

**Table 5. Intention of using HIV self-testing in the future among 170 men who have sex with men: ordered logistic regression***

| | **Univariate analysis** | | **Multivariable analysis**** | |
|---|---|---|---|---|
| | **Odds ratio (95% CI)** | **P** | **Adjusted odds ratio (95% CI)** | **P** |
| Full time employment | 0.59 (0.33 – 1.08) | 0.09 | 0.54 (0.30 – 1.00) | 0.05 |
| Born overseas | 3.00 (1.66 – 5.41) | <0.001 | 3.07 (1.42 – 6.64) | 0.005 |
| Taking PrEP | 0.54 (0.30 – 0.97) | 0.04 | 0.55 (0.30 – 1.00) | 0.05 |
| Self-evaluated ease of performing the oral HIV self-testing | 1.15 (1.06 – 1.24) | 0.001 | 1.14 (1.06 – 1.24) | 0.001 |
| Self-evaluated confidence of performing the oral HIV self-testing | 1.11 (1.02 – 1.20) | 0.01 | 1.11 (1.03 – 1.20) | 0.01 |
| Unable to complete the blood-based HIV self-testing process | 0.39 (0.16 – 0.97) | 0.04 | 0.42 (0.16 – 1.06) | 0.07 |
| Self-evaluated ease of performing the blood-based HIV self-testing | 1.23 (1.12 – 1.36) | <0.001 | 1.25 (1.13 – 1.38) | <0.001 |
| Confidence in performing the blood-based HIV self-testing | 1.16 (1.06 – 1.28) | 0.002 | 1.17 (1.06 – 1.29) | 0.002 |

CI = confidence interval; PrEP = pre-exposure prophylaxis.

***** We tested the following variables: age; sexuality; university education (compared to high school or below); full-time employment; born overseas; ethnic background other than European ancestry; amount of time spent with gay/homosexual friends; having more than 6 male partners in last 6 months; frequency of participation in group sex in the last 6 months; frequency of use of condoms in anal intercourse with regular partners; frequency of use of condoms in anal intercourse with casual partners; taking PrEP; frequency of HIV testing; having ever put off doing a HIV test; having previously conducted HIV self-testing; first test being the oral HIV self-test; being unable to complete the oral HIV self-testing process; self-evaluated ease of performing oral HIV self-testing; self-evaluated confidence of performing oral HIV self-testing; observed difficulty of performing oral HIV self-testing; being unable to complete the blood-based testing process; self-evaluated ease of performing blood-based HIV self-testing; self-evaluated confidence of performing blood-based HIV self-testing; observed difficulty of performing blood-based HIV self-testing. Apart from the variables included in the table, all results were statistically non-significant.

*Dependent variables used a Likert scale of participants' willingness to use HIV self-testing or offer HIV self-testing to their sexual partners. Scores ranged from 0-5, with 0 being "very unlikely" and 5 being "very likely".
**Adjusted for age, education level, and ethnic background.

**Table 6. Intention of performing secondary distribution of HIV self-testing among 170 men who have sex with men: ordered logistic regression***

| | Univariate analysis | | Multivariable analysis‡ | |
|---|---|---|---|---|
| | **Odds ratio (95% CI)** | **P value** | **Adjusted odds ratio (95% CI)** | **P value** |
| **Intention of offering HIV self-testing to a regular partner†** | | | | |
| Age | 0.97 (0.94 – 0.99) | 0.008 | | |
| Born overseas | 3.94 (2.19 – 7.07) | <0.001 | 2.76 (1.27 – 5.98) | 0010 |
| Ethnic background other than European ancestry | 3.39 (1.90 – 6.08) | <0.001 | | |
| Taking PrEP | 0.53 (0.30 – 0.93) | 0.027 | 0.61 (0.34 – 1.07) | 0.082 |
| Self-evaluated ease of performing oral HIV self-testing | 1.13 (1.05 – 1.21) | 0.001 | 1.12 (1.04 – 1.21) | 0.003 |
| Self-evaluated confidence of performing oral HIV self-testing | 1.11 (1.03 – 1.19) | 0.008 | 1.11 (1.02 – 1.20) | 0.012 |
| Being unable to complete the blood-based HIV self-testing process | 0.33 (0.13 – 0.83) | 0.018 | 0.43 (0.17 – 1.09) | 0.075 |
| Self-evaluated ease of performing blood-based HIV self-testing | 1.22 (1.11 – 1.33) | <0.001 | 1.23 (1.12 – 1.35) | <0.001 |
| Self-evaluated confidence of performing blood-based HIV self-testing | 1.16 (1.06 – 1.28) | 0.001 | 1.17 (1.07 – 1.29) | 0.001 |
| **Intention of offering HIV self-testing to a casual partner†** | | | | |
| Sexuality (gay) | 0.42 (0.20 – 0.89) | 0.023 | 0.43 (0.20 – 0.93) | 0.031 |
| Born overseas | 3.53 (2.00 – 6.25) | <0.001 | 3.37 (1.55 – 7.35) | 0.002 |
| Ethnic background other than European ancestry | 2.63 (1.50 – 4.62) | 0.001 | | |
| More than six male partners in last six months | 0.37 (0.21 – 0.64) | <0.001 | 0.42 (0.24 – 0.75) | 0.003 |
| Self-evaluated ease of performing oral HIV self-testing | 1.08 (1.01 – 1.15) | 0.028 | 1.07 (1.00 – 1.15) | 0.047 |
| Being unable to complete the blood-based HIV self-testing process | 0.28 (0.11 – 0.71) | 0.007 | 0.35 (0.14 – 0.90) | 0.030 |
| Self-evaluated ease of performing blood-based HIV self-testing | 1.17 (1.08 – 1.27) | <0.001 | 1.17 (1.08 – 1.28) | <0.001 |
| Self-evaluated confidence of performing blood-based HIV self-testing | 1.10 (1.01 – 1.19) | 0.036 | 1.10 (1.01 – 1.20) | 0.030 |
| Observed level of difficulty of performing blood-based HIV self-testing | 0.85 (0.76 – 0.95) | 0.004 | 0.87 (0.78 – 0.98) | 0.016 |

***** We tested the following variables: age; sexuality; university education (compared to high school or below); full-time employment; born overseas; ethnic background other than European ancestry; amount of time spent with gay/homosexual friends; having more than 6 male partners in last 6 months; frequency of participation in group sex in the last 6 months; frequency of use of condoms in anal intercourse with regular partners; frequency of use of condoms in anal intercourse with casual partners; taking PrEP; frequency of HIV testing; having ever put off doing a HIV test; having previously conducted HIV self-testing; first test being oral HIV self-testing; being unable to complete the oral HIV self-testing process; self-evaluated ease of performing oral HIV self-testing; self-evaluated confidence of performing oral HIV self-testing; observed difficulty of performing oral HIV self-testing; being unable to complete the blood-based testing process; self-evaluated ease of performing blood-based HIV self-testing; self-evaluated confidence of performing blood-based HIV self-testing; observed difficulty of performing blood-based HIV self-testing. Apart from the variables included in the table, all results were statistically non-significant.

† Dependent variables used a Likert scale of participants' willingness to use HIV self-testing or offer HIV self-testing to their sexual partners. Scores ranged from 0-5, with 0 being "very unlikely" and 5 being "very likely".
‡ Independent variables adjusted for in the multivariate analysis were age, education level, and ethnicity
